# Changing the stroke network during pandemic scenarios does not affect the management of patients with a positive Cincinnati prehospital stroke scale

**DOI:** 10.1007/s10072-023-07046-7

**Published:** 2023-09-06

**Authors:** Nazzareno Fagoni, Lorenzo Bellini, Rodolfo Bonora, Marco Botteri, Maurizio Migliari, Andrea Pagliosa, Giuseppe Maria Sechi, Carlo Signorelli, Alberto Zoli, Giuseppe Stirparo

**Affiliations:** 1 Department of Molecular and Translational Medicine, University of Brescia, Brescia, Italy; 2 AAT Brescia, Agenzia Regionale Emergenza Urgenza (AREU), ASST Spedali Civili Di Brescia, Brescia, Italy; 3 Faculty of Medicine, School of Public Health, University of Vita-Salute San Raffaele, Milan, Italy; 4 Department of Research and Development, Agenzia Regionale Emergenza Urgenza Headquarters (AREU HQ), Milan, Italy

**Keywords:** Prehospital, Emergency medical services, Cerebral ischemia

## Abstract

**Introduction:**

Time plays a crucial role in the management of stroke, and changing the prehospital emergency network, altering the HUB and spoke relationship in pandemic scenarios, might have an impact on time to fibrinolysis or thrombectomy. The aim of this study was to evaluate the time-dependent stroke emergency network in Lombardy region (Italy) by comparing 2019 with 2020 and early 2021. Three parameters were investigated: (i) time of arrival of the first vehicle at the scene, (ii) overall duration of missions, and (iii) number of patients transported by emergency vehicles.

**Methods:**

Data analysis process conducted using the SAS-AREU portal (SAS Institute, USA).

**Results:**

The number of patients with a positive CPSS was similar among the different pandemic waves. Mission duration increased from a mean time (SD) of 52.9 (16.1) min in 2019 to 64.1 (19.7) in 2020 and 55.0 (16.8) in 2021. Time to first vehicle on scene increased to 15.7 (8.4) min in 2020 and 16.0 (7.0) in 2021 compared to 2019, 13.6 (7.2) (*P* < 0.05). The number of hospital with available stroke units decreased from 46 in 2019 to 10 during the first pandemic wave.

**Conclusions:**

The pandemic forced changes in the clinical mission of many hospitals by reducing the number of stroke units. Despite this, the organization of the emergency system allowed to identify strategic hospitals and thus avoid excessive transport time. The result was an adequate time for fibrinolysis/thrombectomy, in agreement with the guidelines. Coordinated management in emergency situations makes it possible to maintain service quality standards, despite the unfavorable scenario.

## Introduction

Cerebral ischemia is one of the time-dependent diseases that requires integrated management that works seamlessly between prehospital rescue and arrival at specialized hospitals for fibrinolysis (HUB and spoke hospitals) or intra-arterial procedures (HUB).

The pandemic implications for stroke management consist of reduced diagnoses [1]; fewer hospitalization for transient, mild, and moderate strokes [2, 3]; and increased time between the onset of symptoms and arrival in stroke unit [4]. The Lombardy region (Italy) was among the first European areas to be affected by the COVID-19 outbreak [5, 6]. The impact of the pandemic on the emergency medical system (EMS) was important [7–13], and the networks of time-dependent diseases were characterized by significant changes [14–16], in order to optimize the management of hospitals dedicated to COVID-19 patients. EMS in the Lombardy region is led by AREU (Agenzia Regionale Emergenza Urgenza), which coordinates all prehospital emergency missions, through four command centers called SOREU (Sale Operative Regionali dell’Emergenza Urgenza—Regional Emergency and Urgency Operations Room), supervised by a physician who supports the emergency missions. This system ensures that the correct resources are used and that the patient is sent to the proper hospital according to clinical severity [17–19].

Current guidelines suggest the development of triage paradigms and protocols to ensure that patients with suspected stroke are quickly identified and evaluated through the use of screening tools; however, a recent review stated that it is not possible to make a strong recommendation for the use of one tool over another due to the fact that they all inadequately account for false-negative cases [20, 21]. To ensure rapid identification of time-dependent diseases, AREU uses clinical and physiological criteria. In order to identify patients potentially suffering from stroke, the clinical protocol involves the application of the Cincinnati Prehospital Stroke Scale (CPSS) [22, 23], despite the fact that the CPSS has a sensitivity of 44%–95% and poor specificity, ranging from 24 to 79% [21]. If the CPSS is positive, the patient is rapidly transported to a hospital with a stroke unit [24]. Time plays an important role in stroke management. Indeed, as the most recent guidelines show, in case of ischemic stroke, fibrinolytic therapy should be given within 4.5 h, and in case of favorable perfusion imaging profile detected by automated perfusion imaging within 9 h after the onset of stroke or at the time the patient awoke with stroke symptoms [21, 25].

During the pandemic, EMS and emergency departments (EDs) required reorganization: dedicated pathways were added for patients with suspected COVID-19 [26], health professionals in both EDs and EMS had to modify their personal protective equipment (PPE) [27–29], there was an increase in transport time by EMS rescue vehicles [8, 12, 30, 31], and several changes in the time-dependent disease networks [2, 11, 32–34].

However, there is little evidence regarding the post-pandemic phase; it is of paramount importance for all EMS stakeholders to understand whether the patient transport time and the EMS efficiency in the post-pandemic phase are similar to the pre-pandemic standard. This is important to verify the need to implement specific clinical management protocols. Therefore, the aim of this study is to evaluate the time-dependent stroke network in the Lombardy region, comparing 2019 with the different pandemic phases, through three performance parameters: (i) time to first vehicle on scene, (ii) overall duration of missions, and (iii) number of patients transported by emergency vehicles.

## Methods

This is a retrospective observational cohort study. The study was conducted in accordance with the principles of the declaration of Helsinki.

In Italy, consent is not required for clinical emergency management, and all missions are anonymous and recorded. This means that patients never give consent for their data to be stored. However, consent for a study would be different from consent for clinical purposes. The data are needed to provide the emergency service and are used during missions. After the mission, the data is archived without the possibility of recognizing the patient’s first and last name, but each mission is linked to a number. Since this is a retrospective study on current administrative databases, informed consent is not required; instead, the use of the data in the database was requested and released to the AREU Data Protection Officer in July 2021.

### Data registry

The data were provided by the registry of the Lombardy office of AREU. The data analysis process was conducted using the SAS-AREU portal (SAS Institute, Cary, North Carolina, USA). The portal contains all data related to emergency calls, and the scenarios concerning stroke were selected.

In Lombardy, citizen calls 112 for health problems and clinical emergencies. AREU manages the 112 system and all prehospital emergency service [8, 12, 18]. The EMS receiver automatically opens a record in the SAS-AREU portal and enters the demographic characteristics and clinical problem information provided by the patients. In case of a neurological symptom, a basic life support vehicle and/or an advanced cardiovascular vehicle are deployed to the scene. The vehicle is equipped with a GPS tracker and an automatic system that records the rescue time. During the rescue procedure, the operators inform the medical director of SOREU with all other information to define the patient’s clinical status and the correct hospital. All data are recorded in the SAS-AREU database. All data are needed to identify the appropriate HUB hospital, while the GPS tracker automatically records logistical data.

The study analyzed the number of annual diagnoses, the average time to the first vehicle at the scene, and the average time of transport from the scene to the hospital.

### Statistical analysis

Categorical variables are presented as number, and continuous variables are presented as mean and standard deviation (SD). Categorical variables were analyzed by means of χ^2^ test; relative odds ratios (OR) and 95% confidence intervals (CI95%) were provided. *T*-tests for independent variables were used to assess differences between the mean numbers of patients with positive CPSS per month.

Continuous variables were tested for normality using the Kolmogorov–Smirnov test, and the appropriate analysis for unpaired data was applied.

Differences were considered significant when *P* < 0.05; otherwise, they were considered non-significant (NS). The Prism 8.0.1 statistical software (GraphPad Software LLC, San Diego, CA, USA) was used for this purpose.

## Results

The number of patients with positive CPSS was not different between 2019 and other years, nor did it differ between March 2019 and March 2020, which was the month of the first pandemic peak (Table 1).

**Table 1:** Number of patients with positive CPSS per month (identified by the EMS)

Month	2019	2020	2021
January	880	985	985
February	756	907	789
March	804	830	857
April	756	861	885
May	831	866	838
June	781	861	
July	780	779	
August	805	686	
September	821	847	
October	988	925	
November	987	841	
December	1012	935	
Diagnosis/year	10,201	10,323*	4354*
OR March 2020 and 2021 vs 2019		1.05 [0.95–1.16] *P* > 0.05	0.98 [0.88–1.09] *P* > 0.05

^*^t test for independent data versus 2019 (NS)

**Fig. 1:**
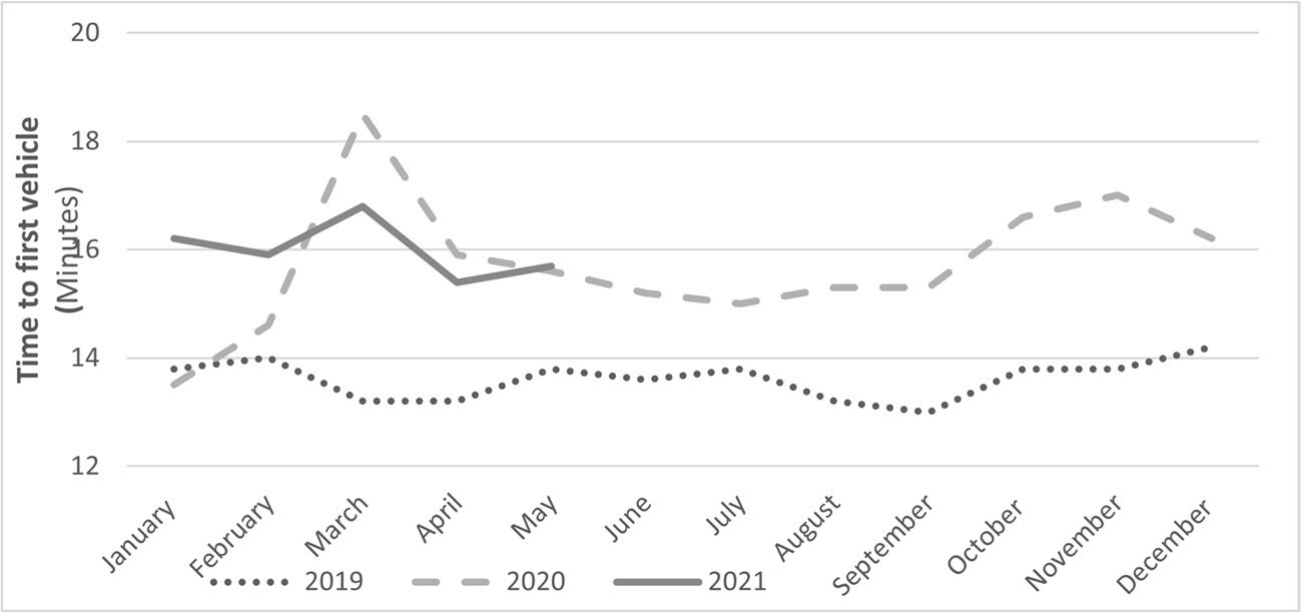
Mean time to first vehicle on the scene for each month

**Fig. 2:**
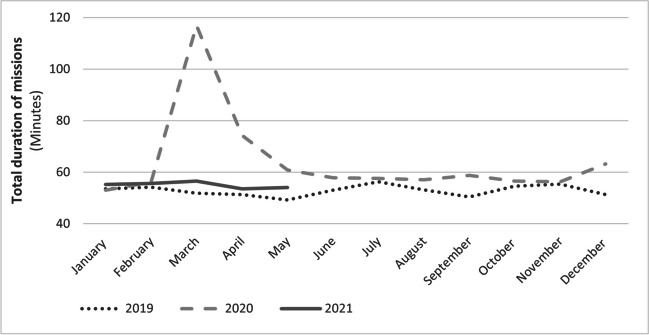
Mean duration of the missions, for each month

Figure 1 shows a significant increase in the mean time (SD) to first vehicle on scene in 2020, 15.7 (8.4) min, and 2021, 16.0 (7.0) min, compared to 2019, 13.6 (7.2) min (*P* < 0.0001 for both years). The largest increase is in March 2020, as shown in Fig. 2.

Comparing 2020 and 2021 with 2019, there is a significant increase in the overall duration of missions, considered as the time from vehicle departure to arrival at the hospital. The time increased from a mean (SD) of 52.9 (16.1) min in 2019 to 64.1 (19.7) min in 2020 and 55.0 (16.8) min in 2021 (*P* < 0.05). The maximum mean time is recorded in March 2020, as shown in Fig. 2, with a peak of 116.9 min, 65.1 min more than the average in 2019.

The median time with its interquartile range, minimum, and maximum is shown in Table 2.

**Table 2:** Medians, IQR, 99° centiles, and ranges for the time to first vehicle on scene and for the time from vehicle departure to arrival at the hospital

	Years	Median (IQR) *min*	99° *min*	Range min–max *min*
Time to first vehicle on scene	2019	12.2 (6.5)	42.4	0.5–125.9
	2020	14.1 (7.1)	46.3	1.2–173.1
	2021	14.2 (6.9)	42.5	1.8–124.1
Time from vehicle departure to arrival at the hospital	2019	43.8 (17.1)	102.7	4.0–272.4
	2020	51.3 (20.6)	117.3	9.5–446.3
	2021	49.9 (18.8)	111.1	12.5–192.2

**Fig. 3:**
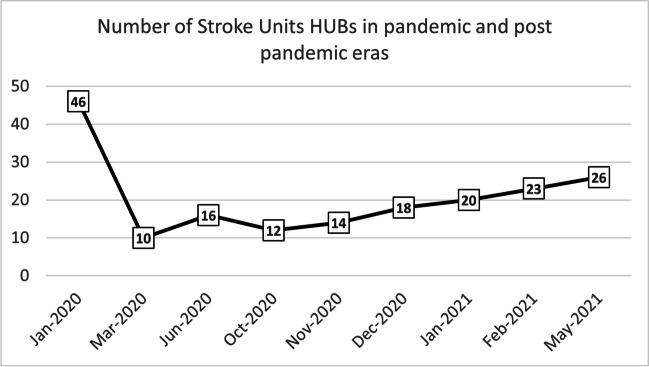
Number of hospitals with a stroke unit in the Lombardy region from the beginning of the outbreak, until May 2021

**Fig. 4:**
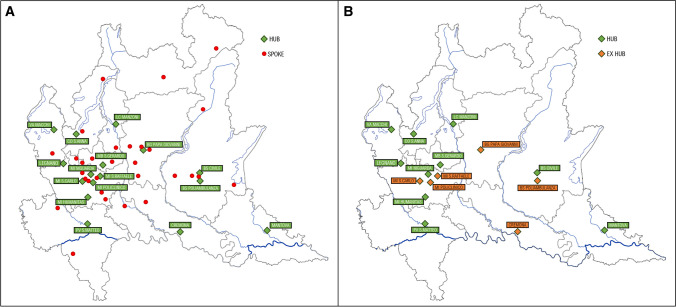
Maps of Lombardy region. **A** Hub and spoke hospital in 2019 before the pandemic. **B** Hubs with effective stroke unit in March 2020 after the beginning of pandemic

Figure 3 shows the number of hospitals to which CPSS-positive patients were referred during the different periods analyzed. After June 2021, the stroke HUB and spoke network was restored to its pre-pandemic organization. Both level 1 and 2 hospitals can perform intravenous thrombolysis, but only the level 2 hospitals can perform endovascular thrombectomy. Figure 4 shows the map of hospitals with stroke unit in 2019 before the pandemic (panel A), consisting of 46 presidia, of which 16 were HUBs and 30 were spokes. Ten HUBs were operative during the pandemic (panel B), whereas 4 HUBs and all spokes were mobilized for COVID-19 patients and did not accept patients with suspected stroke.

## Discussion

The stroke network has undergone profound modifications in several Italian regions [14] as a consequence of the pandemic scenario that swept through the Lombardy region as the first epicenter [35]. Indeed, the reduction in the number of centers with effective stroke unit was established by local health authority in the Lombardy region: from 46 hospitals in the pre-pandemic era to 10 hospitals in the first pandemic wave, and in particular were effective 10 HUBs, while four HUBs were converted, as well as all the spokes.

All the resolutions published by the local health authority were analyzed. A direct impact of these laws was the reduction in the number of hospitals with a stroke unit; this may partly explain the longer overall duration of missions during the pandemic period compared with the previous year.

In the subsequent analysis, the number of patients with positive CPSS did not change in 2020 and 2021 compared to 2019. Although it is not possible to draw parallels between patients with positive CPSS and patients with stroke, given the absence of differences between the pandemic months and 2019, it can theoretically be assumed that the number of patients with stroke was not so different between the years; notwithstanding, it should be noted that in the pandemic months, many patients delayed calling the ambulance, some even avoided contacting the emergency system, and many died at home without knowing the etiology. Aminiti et al. [1] found a reduction in stroke diagnoses in March 2020, and several authors have highlighted this phenomenon [3]. However, they have analyzed all EDs discharge records, where the diagnosis of stroke is made certain by second level exams, such as computerized tomography. In this study, data were based only on a clinical scale, the CPSS. According to De Luca et al. [22], CPSS is a useful tool during referral procedures to identify potential patients with stroke, with high sensitivity but low specificity. In fact, CPSS identified 69 to 90% of strokes, with a sensitivity of 88% to detect strokes of the carotid distribution. However, CPSS fails to identify up to 40% of patients with posterior circulation events, since strokes of the vertebrobasilar distribution occur without CPSS symptoms in 77% of patients [36–39]. During the first wave, the number of presumed strokes detected by EMS personnel using the CPSS did not change; this is to underline that this scale does not consider other parameters or signs that could be altered in the case of a COVID-19-positive patient, so the authors believe that it was a reliable tool even in the presence of a large number of COVID-19 patients.

Although no differences were found in the number of patients rescued with positive CPSS, it is noteworthy that the data noted by Sacco are in line with the reorganization of the emergency system. The fact that only HUBs were retained is probably the cause of the increase in primary endovascular treatments noted by Sacco and colleagues.

For all EMS stakeholders, the analysis of rescue times plays a central role, and this is why this study highlights both the time to first vehicle on scene and the overall duration of mission. The former increased significantly in 2020 (15.7 min) and in 2021 (16.0 min) compared to 2019 (13.6 min).

Figure 2 shows the overall average mission times from the vehicle departure to hospital arrival. A significant increase was observed in 2020 and 2021 compared to 2019. Indeed, in March 2020, during the first pandemic wave, the time for the arrival of the first vehicle at the scene is significantly increased, while thereafter, a consistent delay in the time to first vehicle on scene was recorded, with no further peaks in the second and third waves, in October, November, and December 2020. The consistent delays were likely related to the dressing and undressing times of the EMS operators and the longer travel times of the vehicles, probably related to the same reasons discussed above. However, the stroke chain of survival also consists of intrahospital management, and any delay during these phases would add to the prehospital delay with an impact on clinical outcome. In addition, patients were misinformed to stay away from the hospital during the pandemic, so there was also a delay in calls to the EMS. These aspects were not analyzed in our study, because access to prehospital data does not allow access to information collected at hospitals. Therefore, it should be emphasized as an important limitation of the study.

Despite a significantly longer time to the first vehicle on the scene during pandemic, the most recent guidelines in the case of ischemic stroke suggest that fibrinolysis should be performed within a maximum of 4.5 h from the onset of symptoms, or within 9 h in case of favorable perfusion imaging profile detected by automated perfusion imaging [21], and it is likely that the delay does not affect the time to reperfusion. However, the impact on short-term outcomes and long-term disability might be of great interest for future investigations, since they are not considered in the present study. Figure 3 shows a marked increase in the overall average mission duration during the first pandemic wave. Indeed, in March 2020, the average was 116.9 min, twice as long as in 2019, when 51.8 min was recorded. This increase could have an important clinical impact, compared to the time of the first vehicle on scene: the latter parameter increased 5.3 min in March 2020 compared to 2019, while the overall mission duration increased by 65.1 min. Possibly, vehicles experienced longer delays due to the need to reach hospitals located at greater distances as a result of the reduction in the number of hospitals with stroke unit. However, it should be noted that on average, less than 1% of patients reached the hospital more than 2 h after the call to the emergency system; in contrast, the maximum time reached for transportation to the hospital during the pandemic increased considerably (Table 2), from 272.4 to 446.3 min.

For all EMS stakeholders, overall mission duration is a key parameter in time-dependent disease networks, so AREU implemented organizational measures to prevent peak delays in the second and third waves, as discussed by Marrazzo F. et al. [40] and Spina S. et al. [41].

During the first pandemic wave, EMS came under strong pressure, as demonstrated by Fagoni et al. [12] and by Stirparo et al. [8–10], due to the increase in the number of calls and requests for intervention.

The reorganization of the emergency system was functional to preserve some hospitals for time-dependent conditions, while trying to limit, as was done, possible delays and disruptions in the management of acute patients. The transformation of spoke hospitals into dedicated COVID-19 hospitals was indispensable to try to treat the largest number of people, especially during the first wave that hit Italy and that in fact underscored how there were problems managing such a large number of patients (many critical patients were transferred to Germany and to other regions of Italy). However, this reorganization could not be maintained in the face of excessive efforts: the drastic reduction of hospitals available to accept possible acute stroke patients could not be maintained for a long time, risking overloading those that remained active and oversaturating their beds. Therefore, although the solution proved adequate to address the problem, it could not be maintained as a permanent solution in stroke network management.

### Limitations of the study

A limitation of our study may be the low accuracy in the assessment of the CPSS, despite the fact that the other scales are also considered inadequate because they accounted for false-negative cases (21). In addition, it is not possible to consider out-of-hospital time as the sole actor in the management of the patient with positive CPSS, although it should be noted that AREU provides management protocols that allow for continued in-hospital investigations with priority in patients with suspected stroke, as well as for patients managed within the other time-dependent disease networks.

## Conclusions

The COVID-19 pandemic caused a change in the stroke time-dependent network, with a significant reduction in the number of effective stroke units in the Lombardy region, longer times to the first vehicle on scene, and longer overall mission duration. EMS proved to be resilient during the second wave of COVID-19; in fact, this study did not record another peak in overall mission duration. However, after the pandemic waves, the delay was still present, and this could be explained by the reduction in the number of available stroke units. Coordinated management in emergency situations makes it possible to maintain service quality standards despite the unfavorable scenario.

## Data Availability

The datasets generated during and/or analyzed during the current study are available from the corresponding author on reasonable request.
